# Chronic Subdural Haematoma in the Elderly: Is It Time for a New Paradigm in Management?

**DOI:** 10.1007/s13670-016-0166-9

**Published:** 2016-03-23

**Authors:** J. Shapey, L. J. Glancz, P. M. Brennan

**Affiliations:** National Hospital for Neurology and Neurosurgeon, Queen Square, London, UK; Department of Neurosurgery, Nottingham University Hospitals NHS Trust, Nottingham, UK; Department of Clinical Neurosciences, Western General Hospital, University of Edinburgh, Edinburgh, UK

**Keywords:** Chronic subdural haematoma, CSDH, Elderly, Peri-operative care, Outcomes

## Abstract

Chronic subdural haematoma (CSDH) is a common neurological condition that usually affects the elderly. The optimal treatment strategy remains uncertain, principally because there is a lack of a good evidence base. In this paper, we review the literature concerning the peri-operative and operative care of patients. In particular, we highlight the non-surgical aspects of care that might impact on patient outcomes and CSDH recurrence. We propose that an integrated approach to care in patients with CSDH, similar to care of fragility fractures in the elderly, may be an important strategy to improve patient care and outcomes.

## Introduction

The incidence of chronic subdural haematoma (CSDH) increases with age, and after 70 years of age is 8·2/100,000/year [1]. As the population ages, the prevalence is anticipated to increase [1, 2]. CSDH is an important cause of morbidity and mortality and it has been described as a ‘sentinel health event’ indicating underlying systemic pathology. CSDH has a 1-year mortality similar to that of hip fractures [2].

It is surprising then that little class I evidence exists to describe the optimal surgical and peri-operative management of CSDH. This is important, because improving outcomes in this elderly and often frail population requires us to make informed decisions at all stages of their management, not just in the operating theatre. We need to understand the impact of both surgical and non-surgical treatment decisions on patient outcomes and CSDH recurrence.

The peer-reviewed literature actually offers often conflicting advice regarding CSDH management, leading to considerable variation in practice. For example, the most common surgical strategy for evacuation of a CSDH is burr hole craniotomy, but the optimal number of burr holes (one vs. two) is uncertain [3, 4]. Post-operatively, there is also contradictory advice about the relative risk of bed rest and early mobilisation on CSDH recurrence or post-operative morbidity [5–7]. Other uncertainties include the benefit of corticosteroids as either a primary treatment or an adjunct to surgery and the need to administer agents to mitigate the biological effects of antiplatelet medication pre-operatively (e.g., platelet transfusion) [8, 9]. We will review these in more detail.

Clinical uncertainties create potential risk for elderly patients. A sound evidence base is therefore urgently needed. Crucially, this should focus not only on surgical practice, but should also take into account all stages of the patient pathway from diagnosis to rehabilitation. We review the evidence base for the peri-operative and operative care of patients with CSDH. In particular, we discuss the non-surgical interventions that might facilitate patient recovery and rehabilitation.

## Pathophysiology

CSDH is a collection of liquefied blood between the dura and arachnoid layer of the brain thought to result from injury to bridging veins crossing the subdural space. Head injury is a common risk factor and in a study of 1000 patients, 61.7 % recalled a recent one [10]. Other risk factors include coagulopathy, use of antiplatelet or anticoagulant medication, over-drainage from a cerebrospinal diversion device, haematological malignancies and vascular malformations [11••].

CSDHs often present several weeks or months after the index bleed, because as the initial acute haematoma liquefies it enlarges. This increasing volume causes mass effect that manifests clinically. Two principal theories have been proposed for the mechanism of clot enlargement. Firstly, that the liquefying clot has increased protein content exerting an osmotic effect through increased oncotic pressure. However, the osmolality of the liquefied clot is actually the same as cerebrospinal fluid and blood [12]. A second theory states that recurrent bleeding occurs from abnormal and dilated blood vessels in the capsule that forms around the haematoma. Evidence from radiolabelling studies and observation of coagulation abnormalities within the CSDH itself support this theory [13]. High concentrations of vascular endothelial growth factor (VEGF) have also been demonstrated within the subdural fluid supporting the theory that ongoing angiogenesis and hyper-permeability of capillaries contributes to haematoma expansion [13].

## Clinical Presentation

Patients with chronic subdural haematomas can present in a variety of ways, and symptom onset and progression may range from days to weeks. Elderly patients frequently present with multiple symptoms that may mimic a stroke or rapidly progressive dementia. In a study of 1000 patients with CSDH, their presenting symptoms included behavioural disturbance (28.5 %), headaches (25.1 %), and limb weakness (24.8 %); behavioural disturbance was the predominant clinical feature in elderly patients [10]. Many patients have a mild reduction in their level of consciousness (Glasgow Coma Scale [GCS] score of 13–15), but elderly patients with CSDH do not typically present in coma [14••, 15•]. Bilateral subdural haematomas may be present in up to 25 % of patients, but without causing focal neurological deficits [13, 16]. Midline shift may be minimal with bilateral CSDH, but the haematomas can still exert significant mass effect. This may increase the risk of rapid deterioration, so consideration may be given to expedited surgical drainage in these patients.

## Management

### Surgical Management

Surgical haematoma evacuation is indicated in patients who deteriorate or do not improve. Surgery can bring a rapid clinical improvement with a favourable outcome in over 80 % of patients [1]. However, the most effective surgical technique is uncertain. The three most common techniques are twist-drill craniotomy (TDC), burr hole craniotomy (BHC) and craniotomy. TDC involves making a skull opening of <5 mm and can be performed at the bedside. BHC and craniotomy are performed in the operating theatre; the former involves making one or two holes <30 mm in diameter in the skull, whereas craniotomy is generally defined as creating a >30-mm diameter bony defect which is replaced at the end of the procedure. Several studies including a recent large prospective multi-centre audit of CSDH management in the UK found BHC to be the most frequently used method of surgical drainage [17••, 18•].

#### Comparison of Operational Techniques

Most studies comparing surgical techniques have been small, single-centred and retrospective. Weigel and colleagues performed meta-analyses of the available data. They reported that whilst all three techniques have approximately the same mortality (2–4 %), craniotomy has significantly higher morbidity and TDC has a higher rate of recurrence, suggesting BHC is the preferred technique [19]. A Monte Carlo decision analysis by Lega et al. also favoured BHC over TDC or craniotomy, given its low recurrence and complication rate [20]. Ducruet et al. concluded that TDC should be the first-line treatment for patients who are high-risk surgical candidates with non-septated CSDH, whilst craniotomy is reserved for patients with membranous CSDH or symptomatic recurrence. Ducruet did not specifically compare TDC to BHC, but a more recent meta-analysis by Almenawer et al. found no significant differences between TDC and BHC in mortality, morbidity or recurrence rates [18•]. However, there was a trend towards lower complication rates in patients treated with TDC [18•].

#### Number of Burr Holes

Current evidence then suggests that TDC or BHC should be first-line treatment in the majority of patients with CSDH. The number of burr holes used depends on individual surgeon preference. A meta-analysis of 631 patients failed to demonstrate a significant difference in recurrence rates related to the number of burr holes used, so single BHC appears as good as double BHC [21]. Similarly, a recent large UK audit did not demonstrate number of burr holes as an independent risk factor for CSDH recurrence [17••]. Perhaps the default surgical method should be a single burr hole, reducing operating time and potentially morbidity. Crucially, this may also permit the procedure to be performed under local anaesthetic, an attractive option in patients with cardio-respiratory morbidity. This hypothesis needs to be formally tested.

#### Irrigation

During surgical evacuation of a CSDH, opening of the dura to release the haematoma often reveals that the collection was under tension. This release of pressure may be sufficient to improve the patient’s clinical state and the role of additional intra-operative irrigation remains unclear. One study of 247 patients demonstrated a significant reduction in recurrence rates when more than 1500 ml of irrigation fluid is used [22]. However, a recent meta-analysis demonstrated no clear evidence of improvement in outcomes [18•].

#### Use of Post-operative Drains

The most evidence-based adjunct to surgical haematoma evacuation is placement of a post-operative drain. A randomised controlled trial (RCT) demonstrated that subdural drains left in situ after BHC reduced CSDH recurrence requiring re-drainage (9·3 vs. 24·0 % recurrence with/without drain) [14••]. The benefit of drains was confirmed by three recent meta-analyses, and the placement of closed-system drainage is now standard practice [18•, 23, 24]. Some surgeons prefer placement of a less-invasive subperiosteal drain and it has been recommended that subperiosteal drains are placed in patients over 80 years of age or in those with predicable high risk for complications [25]. A direct comparison between subdural and subperiosteal drain placement has yet to be made.

### Non-surgical Management

In patients with mild symptoms such as minor headache, or in patients deemed unfit for surgery, there may be a role for ‘watchful waiting’. Serial cranial imaging may provide information to support decision-making, but the success or otherwise of this strategy should be guided by clinical assessment. As yet, there are no evidence-based guidelines on the frequency of serial imaging.

#### Corticosteroids

Some surgeons recommend a course of corticosteroids. Since spontaneous resolution of CSDH can occur, it is difficult to know whether corticosteroids speed up this process. Spontaneous resolution is thought to occur when the vascular membrane around the haematoma matures, bringing an end to the recurrent small bleeding that increases or maintains the size of the haematoma. Corticosteroids may facilitate this process by down-regulating the inflammatory reaction that is thought to maintain the vascularised haematoma membrane [26]. The hypothesis that corticosteroids negate the need for surgery in some symptomatic patients and reduce the rate of recurrence in other surgically treated patients is principally derived from non-randomised studies [8, 25]. However, two prospective RCTs currently recruiting aim to definitively clarify the role of corticosteroids. Dex-CSDH is a multi-centre randomised, double blind, placebo-controlled trial of a 2-week course of tapering dexamethasone for adults with symptomatic CSDH (ISRCTN 80782810). This will assess the clinical status of the patients 6 months post-randomisation through objective disability scales, whilst also establishing progression to surgical intervention and recurrence rates in each group. The Dresh study is a double blind, placebo-controlled RCT [27]. It differs from Dex-CSDH in that the medication is prescribed post-operatively for 6 days. The end point is reoperation rates at 12 weeks.

#### Angiotensin Converting Enzyme Inhibitors

It has been hypothesised that the concurrent treatment of hypertension with angiotensin converting enzyme (ACE) inhibitors in patients with CSDH might lower the risk of recurrence after surgery. ACEI might also lower the risk of developing CSDH through their antiangiogenic mechanism [28]. A trial to assess whether treatment with an ACE inhibitor for 3 months after surgical evacuation of CSDH will decrease the risk of recurrence is ongoing (NCT00915928).

### Peri-operative Care of Elderly Patients with CSDH

The 2001 National Service Framework (NSF) for older people recognised that care of the elderly patient in hospital is complex [29]. It recommends that older people be given early supervision and advice from a specialist team on hospital admission. The increased risk of peri-operative complications with advancing age in part reflects a higher incidence of coexisting disease and polypharmacy.

#### Anticoagulation and Antiplatelet Therapy

In a recent national UK audit of CSDH management, 43 % of patients were admitted on an anticoagulant or antiplatelet medication, similar to previously published figures [30]. Anticoagulant and antiplatelet use are both implicated in CSDH development and recurrence [31]. Warfarin may increase the risk of CSDH development up to 42.5 times [30]. The increased risk from aspirin appears less [30]. The relative risk of developing a CSDH whilst taking newer antiplatelet medications has not yet been studied. The correct peri-operative management of anticoagulant and antiplatelet medication is therefore extremely important.

#### Anticoagulant Medication

The required speed of reversal of anticoagulant and antiplatelet effects to minimise intra- and post-operative bleeding complications will partly depend on the clinical urgency of surgery. Vitamin K usually provides a more gradual normalisation of the international normalised ratio (INR), but may be used in conjunction with fresh frozen plasma (FFP), prothrombin complex concentrate (PCC) or recombinant factor VIIa (rfVIIa) to speed up reversal and avoid INR rebound [32]. FFP can promote fluid overload in elderly patients and may precipitate cardiac or renal impairment, so PCC may be preferred as an adjuvant to vitamin K [32, 33].

Resumption of anticoagulation therapy following surgery should be done cautiously, but the risk of recurrent haemorrhage must be weighed against increased risk of thromboembolic complications with prolonged discontinuation of anticoagulation. There is little good quality evidence in this area. There is a suggestion that oral anticoagulation may be recommended 3 days after surgery in patients with a high thromboembolic risk (e.g., patients with mechanical heart valves) [34]. The CHA2DS2-VASc thromboembolism risk score and the HAS-BLED bleeding risk score may help inform the timing in patients with atrial fibrillation [35].

#### Antiplatelet Medication

The effects of aspirin last until there is a 20 % new circulating platelet mass, which usually occurs 5 days after cessation of medication [36]. Clopidogrel produces abnormal bleeding for approximately the same duration after discontinuation [37]. Anecdotally, in patients with minor symptoms, surgery is therefore usually delayed for 7–10 days. However, the only study to directly examine the effects of delaying surgery on the recurrence rates of CSDH found surgery had to be delayed for only 3 days after stopping antiplatelet medications to reduce risk of recurrence [38]. Reducing the length of time patients wait for surgery may reduce other peri-operative morbidity. For emergency surgery, the American Society of Haematology recommends that patients previously taking aspirin or clopidogrel should be given a pre-operative platelet transfusion [39]. However, these recommendations do not pertain specifically to CSDH and there is no direct evidence that platelet transfusion positively influences outcomes for patients with CSDH.

Newer antiplatelet agents include a direct thrombin inhibitor (Dabigatran), an indirect Factor Xa inhibitor (Fondaparinux), and direct Factor Xa inhibitors (Rivaroxaban and Apixaban). Factor VIII Inhibitor Bypassing Activity (FEIBA) should be the first-line reversal agent in patients taking dabigtran [37]. PCC should be first-line in patients taking rivaroxaban and rfVIIa for those on fondaparinux [37]. The effect of these antiplatelet and reversal agents on incidence of CSDH and outcomes from surgery is not yet known.

There is no quality evidence to guide the optimal timing of post-operative resumption of antiplatelet therapy in patients treated surgically for CSDH. Further prospective studies are needed to provide evidence-based recommendations.

#### Choice of Anaesthesia

A small number of studies have examined the influence of local (LA) and general anaesthesia (GA) on surgical CSDH evacuation. A retrospective study of patients over 60 years old undergoing BHC for CSDH demonstrated that cardiac complications were significantly higher in the GA group, who consequently had significantly longer hospital stays [40]. However, in another single-centre retrospective series of 1000 surgically treated patients, there was no significant difference in outcome between ‘monitored anaesthesia’ (LA with conscious sedation) and GA, although only 10 % of patients in this study actually underwent GA [10].

Comparisons of GA and regional/local anaesthesia have been made in non-neurosurgical practice. For example, a review of 18,715 geriatric patients undergoing hip fracture surgery concluded that spinal anaesthesia is associated with significantly reduced early mortality and a decreased risk of venous thromboembolism, delirium, myocardial infarction, pneumonia and post-operative hypoxia [41]. However, the GALA trial (general anaesthesia versus local anaesthesia for carotid surgery) failed to demonstrate a difference in outcomes (stroke, myocardial infarction or death at 30 days) between GA and LA in patients undergoing carotid surgery [42]. Interestingly, data from patients in the UK national audit of CSDH suggests that many have similar baseline characteristics to those included in the GALA trial. For now, the anaesthetist and surgeon, in consultation with the patient, must decide which anaesthetic technique to use on an individual basis. In the future, if it can be demonstrated that a single TDC or BHC with subdural drain is as effective as more extensive procedures, then the tolerability and acceptability of LA may increase.

#### Time to Surgery

‘Elective & Emergency Surgery in the Elderly: An Age Old Problem’, published in 2010 by the National Confidential Enquiry into Patient Outcome and Death (NCEPOD) highlighted the increased risk of peri-operative morbidity and mortality in the elderly (http://www.ncepod.org.uk/2010eese.htm). The report demonstrated that delays in surgery are associated with poorer outcome and recommends that senior clinicians in surgery, anaesthesia and medicine need to be involved in the decision to operate on elderly patients. Integrated patient care pathways can consolidate the involvement of these specialities to optimise care. For example, these pathways are now the standard of care for NHS patients presenting with a fractured neck of femur and have been shown to reduce morbidity and mortality as well as hospital length of stay [43]. The British Orthopaedic Association and the British Geriatrics Society also provide best practice guidelines in their ‘Blue Book’ publication [44]. Together with a Web-based National Hip Fracture Database (NHFD), this permits trauma units to benchmark and improve their management of this patient population.

No such guidelines or resources exist for elderly patients with CSDH and there is currently no evidence to guide best practice on timing of surgery. Instinctively, the shorter the duration of a neurological deficit, the quicker will a patient’s recovery be and the more likely the deficit is to fully recover. The relative merits of early surgery need to be weighed against the benefits of optimising the patient’s medical state for anaesthesia. A multi-disciplinary integrated patient care pathway to facilitate this is currently under development by The National Hospital, London.

#### Post-operative Mobilisation

Early post-operative mobilisation of patients remains a controversial issue. It is thought to reduce incidence of complications such as pneumonia and deep vein thromboses. Conversely, bed rest has been proposed to reduce recurrence by promoting brain re-expansion. A recent audit of UK practice found that 61 % of practitioners continue to prescribe bed rest for a median of 12–24 h post-operatively, but the same study also demonstrated that bed rest did not significantly affect the rate of CSDH recurrence. In fact, bed rest predicted poor outcomes (mRS 4–6) [17••]. Two prospective randomised studies have also concluded that recurrence rates after BHC are independent of patient’s post-operative position, but another demonstrated a statistically higher recurrence rate in patients mobilised immediately after surgery [5–7]. Reported complication rates as a result of early mobilisation also vary between these studies. Intuitively, if early mobilisation does not impact CSDH recurrence, then it should be beneficial to patient recovery.

## Conclusion: Time for a New Paradigm in CSDH Management—an Evidence-Based Integrated Care Pathway

We have demonstrated that many uncertainties remain as how to best optimise operative and peri-operative care for elderly patients with CSDH. Parallels to patients with fragility fractures would suggest that we need an integrated, multi-disciplinary approach, minimising delay to surgery, simplifying the surgical approach and promoting early rehabilitation. A better coordination of multi-disciplinary care should also improve patient satisfaction in their treatment. Further work to establish the standards to be included in an integrated care pathway will need to address several important questions, amongst them: Is there a benefit from early transfer to a neurosurgical unit or from early surgery? Does routine access to acute medical support on admission improve peri-operative care? Is single burr hole drainage and insertion of a subdural drain performed under monitored anaesthesia (local anaesthetic with sedation) non-inferior/superior to multiple burr holes performed under general anaesthetic? Is there an adjunctive role for corticosteroids? Does early post-operative mobilisation improve long term outcomes or increase CSDH recurrence? Addressing these questions requires a coordinated approach and we invite collaboration in our future work as we endeavour to establish best practice for this vulnerable patient population.
